# Harnessing Apple Cell Suspension Cultures in Bioreactors for Triterpene Production: Transcriptomic Insights into Biomass and Triterpene Biosynthesis

**DOI:** 10.3390/ijms26073188

**Published:** 2025-03-29

**Authors:** Xuan Xu, Emmanuelle Cocco, Gea Guerriero, Kjell Sergeant, Samuel Jourdan, Jenny Renaut, Jean-Francois Hausman, Sylvain Legay

**Affiliations:** Luxembourg Institute of Science and Technology, Technology, 5, Rue Bommel, L-4940 Hautcharage, Luxembourg; emmanuelle.cocco@list.lu (E.C.); gea.guerriero@list.lu (G.G.); kjell.sergeant@list.lu (K.S.); samuel.jourdan@list.lu (S.J.); jenny.renaut@list.lu (J.R.); jean-francois.hausman@list.lu (J.-F.H.); sylvain.legay@list.lu (S.L.)

**Keywords:** plant cell suspension culture, bioreactor, triterpene, coumaroyl-tormentic acid, transcriptomics, upscaling, elicitation, biomass yield, apple

## Abstract

Plant cell suspension cultures offer a sustainable method for producing valuable secondary metabolites, such as bioactive pentacyclic triterpenes. This study established a high-triterpene-yielding cell suspension culture from the apple cultivar “Cox Orange Pippin”. Through transcriptomic analysis and triterpene profiling across growth phases, we uncovered complex regulatory networks that govern biomass production and triterpene biosynthesis. Key biological processes, including cell cycle regulation, cell wall biosynthesis, lipid metabolism, and stress response mechanisms, play pivotal roles in culture dynamics. Differential gene expression linked to these processes revealed how the culture adapts to growth conditions and nutrient availability at each growth phase. Methyl jasmonate elicitation enhanced phenylpropanoid and flavonoid biosynthesis, along with specific triterpene production pathways, highlighting its potential for optimizing secondary metabolite production. Key enzymes, such as oxidosqualene cyclase 4 and a putative C-2α hydroxylase, were identified as promising targets for future metabolic engineering efforts. This study represents the first in-depth report on the molecular mechanisms underlying plant cell growth in bioreactors, specially focusing on a cell suspension culture derived from a semi-russeted apple cultivar. The findings reveal key regulatory pathways in biomass accumulation and triterpene production, offering valuable insights for optimizing bioreactor cultures for industrial applications.

## 1. Introduction

Plant cell suspension cultures have emerged as a promising platform for the sustainable production of valuable secondary metabolites, such as pentacyclic triterpenes, which possess a broad spectrum of bioactive properties [1]. Traditionally, these compounds have been extracted from whole plants; however, this approach faces several challenges, including slow growth rates, environmental impacts, low yields, and seasonal variations. In contrast, plant cell suspension cultures offer a controlled, scalable, and efficient system for producing bioactive compounds, effectively addressing constraints associated with traditional extraction methods [1]. Notable industrial-scale successes include paclitaxel, an anticancer compound produced using Taxus cell cultures [2], and shikonin, a naphthoquinone derivative with pharmaceutical and cosmetic applications derived from *Lithospermum erythrorhizon* cell cultures [3]. These demonstrate the commercial viability of plant cell culture technology, highlighting its potential as a reliable platform for producing bioactive compounds with significant industrial value.

The cell cycle, cell wall composition, and lipid metabolism are fundamental determinants of biomass accumulation and secondary metabolite production in plant cell suspension cultures within bioreactors. The regulation of the cell cycle governs cell division and expansion, directly influencing biomass yield [4]. Meanwhile, the structural integrity and flexibility of the cell wall play a critical role in nutrient uptake and stress responses [5], both of which are essential for optimal growth in bioreactor environments. Lipids, as key components of cell membranes and signaling molecules, regulate cellular processes and secondary metabolite biosynthesis, further impacting culture performance [6]. A comprehensive understanding of these biosynthetic pathways is essential for optimizing bioreactor conditions, allowing for the strategic manipulation of culture parameters to enhance both biomass productivity and metabolite synthesis.

Additionally, the application of elicitors, such as methyl jasmonate (MeJA), represents a highly effective strategy for boosting secondary metabolite production in these cultures [7,8]. Elicitors induce various stress responses within plant cells, activating signaling pathways that enhance metabolite synthesis and accumulation, leading to increased productivity in a controlled cultivation environment [9].

Among the myriad cultivated plant species worldwide, the apple (*Malus* × *domestica*) stands out as one of the most widely grown fruit crops. This remarkable fruit is rich in various phytochemicals, with pentacyclic triterpenes constituting a notable category [10]. These triterpenes represent a significant portion of the epicuticular waxes, accounting for approximately 32–70% depending on the cultivar [11]. Furthermore, it can amount to as much as 60 mg per individual apple [10]. Triterpenes in plants serve vital protective functions against environmental stresses [12]. These compounds also exhibit significant bioactive properties, including anti-inflammatory effects [10], anti-plasmodial activity [13], and anticancer effects [14]. Such diverse properties have garnered significant interest from the pharmaceutical, nutraceutical, and cosmetic industries, highlighting the potential of apple-derived triterpenes in developing functional health products [15].

In most commercially available apple varieties, the predominant triterpenes are ursolic and oleanolic acids [16]. Notably, research has shown that the suberized skin tissue of heritage apple varieties, particularly the partially (such as “Cox Orange Pippin”) and fully russeted types, contains elevated concentrations of lupane derivatives, including betulinic acid and its conjugated form, betulinic acid-3-trans-caffeate, which are found in higher amounts compared to waxy-skinned [17,18].

The biosynthesis of triterpenes initiates with the cyclization of 2,3-oxidosqualene, a 30-carbon precursor sourced from the isoprenoid pathway [19]. This process is catalyzed by oxidosqualene cyclases (OSCs) and occurs in a chair–chair–chair conformation, generating the dammarenyl carbocation, which leads to the formation of triterpenes such as ursane, oleanane, and lupane [15]. Subsequently, these triterpene scaffolds undergo further modifications by various tailoring enzymes, including cytochrome P450s, which introduce functional groups (e.g., aldehyde, hydroxyl, ketone,) at specific positions like C-28. These modifications result in the production of triterpene acids such as oleanolic acid (OA), ursolic acid (UA), and betulinic acid (BA) [20].

While extensive research has focused on the molecular characteristics and triterpene profiling of various apple tissues, such as flesh, skin, and leaves, there have been no similar studies on cell suspension culture systems derived from apple. Furthermore, despite the growing interest in utilizing plant cell suspension cultures to produce valuable compounds, the molecular mechanisms governing biomass production and secondary metabolite biosynthesis remain poorly understood. We developed and scaled up a cell suspension culture derived from the apple cultivar “Cox Orange Pippin” in bioreactors to produce triterpenes. Triterpene profiling was conducted in parallel with a comprehensive transcriptomic analysis of the cell suspension cultures across various growth phases, revealing the molecular processes that regulate both biomass production and triterpene biosynthesis. Additionally, the effect of elicitation with MeJA was examined to explore potential strategies for enhancing the production of secondary metabolites.

## 2. Results and Discussion

### 2.1. Development of Cell Suspension Cultures in Shake Flasks

**Figure 1:**
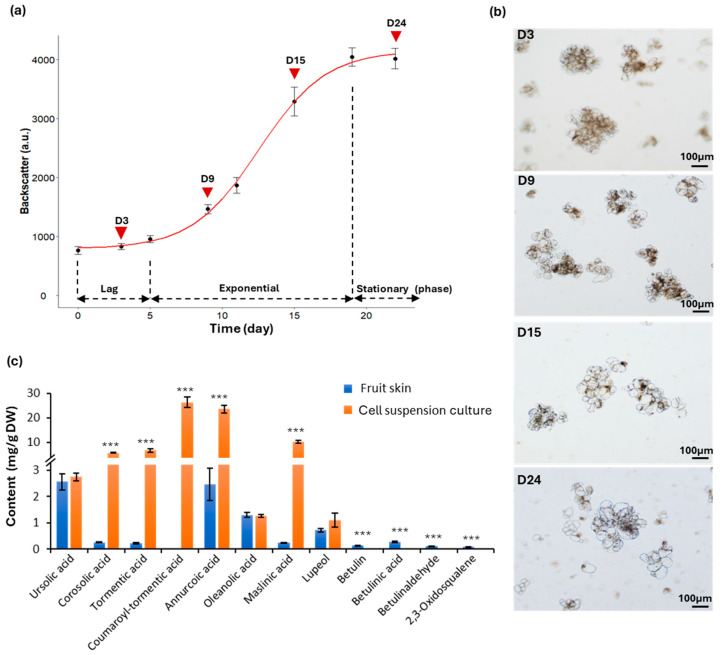
Growth kinetics and triterpene profiling of Cox cell suspension cultures grown in shake flasks. (**a**) The growth kinetics of the cell suspension cultures were monitored in shake flasks with a 20% (*v*/*v*) inoculum. The backscatter value of cultures was recorded every 2–3 days using cell growth quantifier. The values were plotted as the mean ± SD of six biological replicates. Arrowheads indicate the time points (i.e., day 3, 9, 15 and 24) corresponding to the light microscopy analysis displayed in (**b**). (**c**) Triterpene profiles were compared between the cell suspension cultures and the fruit skin. Data represent the mean ± SD of three biological replicates, and statistical significance was assessed using Student’s *t*-test (***, *p* < 0.001).

After 5 weeks, friable calli appeared on the surface of flesh explants. Young and healthy calli exhibited a bright yellow color (Figure S1a) and tended to turn brownish as they grew older. Cell suspension cultures derived from “Cox Orange Pippin” (hereafter referred to as Cox) exhibited a yellowish color, with fine suspended cells and visible aggregates that settled at the bottom of the flask (Figure S1b). The biomass of suspended cells in six shake flasks, with a total culture volume of 100 mL, was monitored by measuring cell densities every 2 or 3 days using a cell growth quantifier (CGQ, Aquila Biolabs). As illustrated in Figure 1a, the lag phase lasted for 5 days, followed by an exponential phase that concluded on day 20. Afterward, the cells entered the stationary phase, where no further growth occurred. The total dry weight of the cell suspension cultures collected on day 24 was 1.2 ± 0.12 g. Microscopic examination of the cultures at different phases (i.e., day (D) 3, 9, 15, and 24) revealed that most cells had a regular ovoid shape and formed multicellular aggregates (Figure 1b). Notably, cells during the log phase (D3) and early-exponential phase (D9) were smaller compared to those in the late-exponential (D15) and stationary phases (D24), indicating cell expansion as they approached the stationary phase, similar to the behavior observed in suspension cultures from other plant species, such as thale cress [21] and tobacco [22].

The triterpene profile of the established cell suspension cultures was analyzed using a targeted UPLC-DAD approach (Figure 1c), and the results were compared with the triterpene content of the fruit skin from the same cultivar. Remarkably, the cell cultures exhibited a significantly higher triterpene content (77.47 ± 4.76 mg/g DW), more than 9-fold greater than the levels found in the fruit skin (8.30 ± 0.94 mg/g DW). Additionally, there were notable differences in the triterpene composition between the cells and the fruit skin. More specifically, in the fruit skin, the dominant triterpenes were ursolic acid (2.56 ± 0.31 mg/g DW), annurcoic acid (2.46 ± 0.61 mg/g DW), oleanolic acid (1.28 ± 0.10 mg/g DW), and lupeol (0.71 ± 0.07 mg/g DW), along with smaller amounts of other triterpenes, including corosolic acid, tormentic acid, maslinic acid, betulin, betulinic acid, betulinaldehyde, and 2,3-oxidosqualene. This triterpene profile aligns with findings from previous studies [17].

In contrast, the most abundant triterpenes in the cell suspension cultures were coumaroyl-tormentic acid (26.34 ± 2.2 mg/g DW) and annurcoic acid (23.54 ± 1.62 mg/g DW), followed by and maslinic acid (10.17 ± 0.49 mg/g DW), tormentic acid (6.66 ± 0.70 mg/g DW), and corosolic acid (5.66 ± 0.06 mg/g DW). Interestingly, neither α-amyrin nor β-amyrin was detected in the extracts from either the cell cultures or the fruit skin. Additionally, triterpenes such as betulin, betulinic acid, betulinaldehyde, and 2,3-oxidosqualene, which were present in the fruit skin, were absent in the cell cultures. Conversely, coumaroyl-tormentic acid, the most abundant triterpene in the cell cultures, was not detected in the fruit skin.

To the best of our knowledge, coumaroyl-tormentic acid has been identified in only a few plant species, including *Vitellaria paradoxa* [23] and *Eriobotrya japonica* [24,25]. The concentration of coumaroyl-tormentic acid in Cox cell suspension cultures is over 131- fold higher than that found in extracts from *Eriobotrya japonica* leaves (0.2 mg/g DW) and more than 9-fold higher than in calli (2.9 mg/g DW) [24]. Several studies have highlighted the remarkable biological properties of coumaroyl-tormentic acid, including its significant in vitro antitrypanosomal activity [23,26], its anti-tumor effects on human oral cell lines [24], and its ability to induce apoptotic cell death in human leukemia cell lines [25].

In addition to coumaroyl-tormentic acid, various triterpenes isolated from *Eriobotrya japonica* leaves have demonstrated bioactivities such as anti-melanogenesis, anti-acne, anti-allergy, anti-inflammatory, and anti-aging effects [27,28]. Triterpenes are also well-known for their wound healing properties, making them promising candidates for the development of new drugs to treat skin injuries [29]. These combined bioactivities underscore the potential of triterpenes as functional ingredients in foods and cosmetics, contributing to overall health.

Collectively, the cell suspension culture developed in this study, rich in triterpenes, particularly coumaroyl-tormentic acid, offers a reliable and valuable source of bioactive compounds. This holds significant potential for a range of industrial applications.

### 2.2. Upscaling in Bioreactor and Triterpene Analysis of Cells

**Figure 2:**
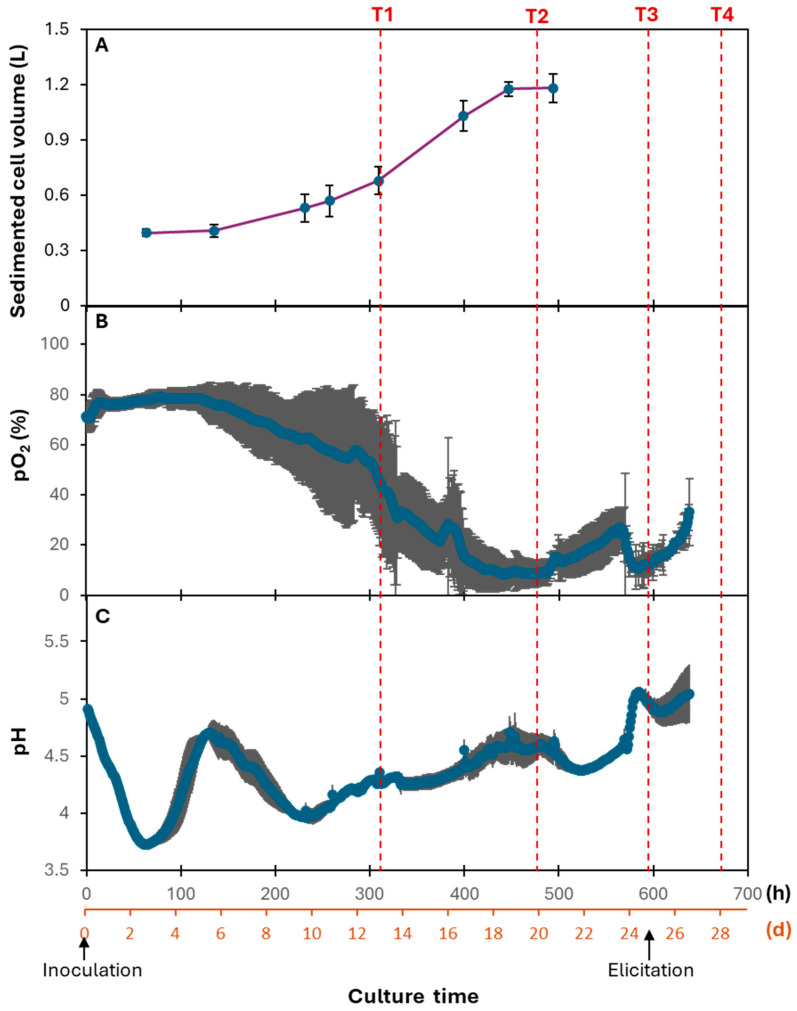
Upscaling “Cox Orange Pippin” cell suspension cultures in bioreactors. Data obtained for (**A**) sedimented cell volume, (**B**) partial pressure of oxygen (pO_2_), and (**C**) pH are represented as averages from three bioreactors and standard deviations are shown as error bars. No sedimented cell volume was recorded after 21 days due to the difficulties in obtaining accurate results from the high-density cell culture. Four time points were identified to represent different growth phases of the cell suspension culture, namely the early-exponential phase (T1), late-exponential phase (T2), stationary phase (T3), and three days after elicitation (T4).

The cell suspension culture was scaled up from flask cultures to a 6 L bioreactor system to examine its growth kinetics and triterpene production at a larger scale (Figure S1c). Biomass accumulation in the bioreactors was monitored using the SCV method. The growth kinetics followed a similar pattern to flask cultures (Figure 2), with a 5-day lag phase, followed by an exponential growth phase that lasted until day 20. After this, the cells entered the stationary phase, where growth ceased. No SCV was recorded after 21 days due to the difficulties in obtaining accurate results from the high-density cell culture. The growth kinetics was consistent with the observed oxygen consumption under a constant air flow rate: during exponential growth, partial oxygen pressure (pO_2_) dropped to 9% and then increased again as the cells transitioned into the stationary phase.

Additionally, the changes in pH throughout the culture were intricately linked to different growth phases. After inoculation, the pH dropped sharply, then increased to 4.6 during the lag phase. It declined to around 4 during the early-exponential phase and gradually increased again as the culture progressed through exponential growth, followed by a slight decrease as the cells entered the stationary phase. These pH fluctuations, along with changes in dissolved oxygen level, reflect the metabolic activity of the cells at each growth stage.

Given the optimal timing for transcriptomic analysis (as explained below), samples collected at four key time points were selected for further investigation: day 13 (T1, early-exponential phase), day 20 (T2, late-exponential phase), day 25 (T3, stationary phase), and 3 days after MeJA elicitation, corresponding to day 28 (T4). This approach facilitated the comprehensive analysis of triterpene accumulation and gene expression patterns across different growth phases, and in response to elicitation.

The dry and fresh weights were assessed at the end of the batch from three bioreactors. Following four 50 mL sampling points taken during the time course, the final measurements yielded a fresh weight of 183.9 ± 11.9 g/L and a dry weight of 10.4 ± 0.7 g/L.

**Figure 3:**
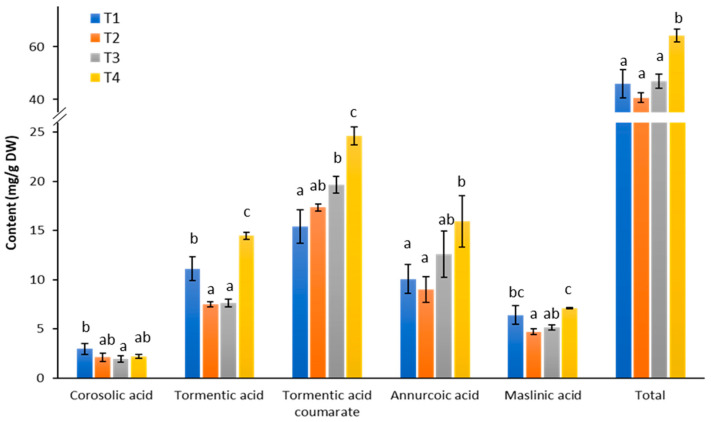
Triterpene profiles of cell suspension cultures at different growth phases and after MeJA elicitation. T1, early-exponential phase; T2, late-exponential phase; T3, stationary phase; and T4, three days after the elicitation with methyl jasmonate. Significant differences among growth phases were analyzed using one-way ANOVA with Tukey’s post hoc test and are indicated with letters.

To investigate triterpene production during cell growth and in response to elicitation, we quantified various triterpenes in cell cultures collected at different time points. The analysis focused on the predominant triterpenes present in the culture (Figure 1), including corosolic acid, tormentic acid, tormentic acid coumarate, annurcoric acid, and maslinic acid. As shown in Figure 3, the total triterpene content remained relatively stable throughout the cell growth period (T1–T3). However, a significant increase in triterpene levels was observed three days after elicitation (T4).

More specifically, at T4, the total triterpene content increased by 40%, 58%, and 37% compared to T1, T2, and T3, respectively. Additionally, most triterpenes showed significantly higher accumulation after elicitation compared to at least one of the growth phases, with the exception of corosolic acid. Notably, tormentic acid levels nearly doubled after elicitation compared to T2 and T3, while the content of tormentic acid coumarate was 60% higher at T4 than at T1. Interestingly, tormentic acid levels declined significantly during the cell growth phase, accompanied by a simultaneous increase in coumaroyl-tormentic acid, suggesting a rapid conversion of tormentic acid to its coumarate form during this period.

This study demonstrated that eliciting cells during stationary phase with 100 µM MeJA is an effective strategy for enhancing triterpene production in Cox cell suspension cultures. Previous research has shown that MeJA concentration and the timing of elicitation (i.e., the specific growth stage of the cell suspension cultures) significantly influence both biomass and secondary metabolite production in plant cell cultures [30,31]. Moreover, the metabolic response of suspension-cultured cells to different elicitors is known to vary greatly [32,33,34,35]. This variability is exemplified by the work of [36], who summarized different elicitation strategies applied to *Panax ginseng*, emphasizing the critical role of elicitor type, concentration, timing, and duration in ginsenoside accumulation and heterogeneity. In light of these findings, further studies on elicitation strategies, such as fine-tuning MeJA-mediated elicitation parameters (duration, concentration, or growth stage) or exploring the use of alternative elicitors in Cox cell suspension cultures, are essential for optimizing triterpene production.

### 2.3. Transcriptomic Profiles of Cell Suspension Cultures Across Time Points

To better understand the molecular mechanisms underlying triterpene production and the effects of elicitation, transcriptomic profiling of the Cox cell suspension cultures was conducted at different time points. Previous transcriptomic studies in plant cell cultures have demonstrated growth phase-dependent shifts. For instance, poplar cell suspension cultures exhibit pronounced transcriptomic changes after the early-exponential phase [37], while *Arabidopsis* cell cultures show major shifts in gene expression during the transition from the late-exponential to stationary phase [38]. Guided by these findings, as well as cell growth data obtained from bioreactor experiments (Figure 2), we selected three time points for the transcriptomic analysis: the early-exponential phase (T1), the late-exponential phase (T2), and the stationary phase (T3). Additionally, to investigate the molecular response to MeJA elicitation in relation to triterpene biosynthesis, we included a fourth time point (T4), three days after MeJA elicitation. This approach enabled us to capture both the growth phase-specific gene expression patterns, and the changes induced by elicitation in the context of triterpene biosynthesis.

RNA-Seq analysis performed on the cells collected at T1, T2, T3, and T4 revealed a total of 8923 differentially expressed contigs with the selected threshold: false discovery rate (FDR) of *p* < 0.05, an RPKM difference greater than |5| and fold change greater than |2| between at least two time points (Supplementary Data S1). The validation of a gene subset using qRT-PCR demonstrated a strong correlation with the RNA-Seq data, with an R^2^ value of 0.96 (Supplementary Data S1).

**Figure 4:**
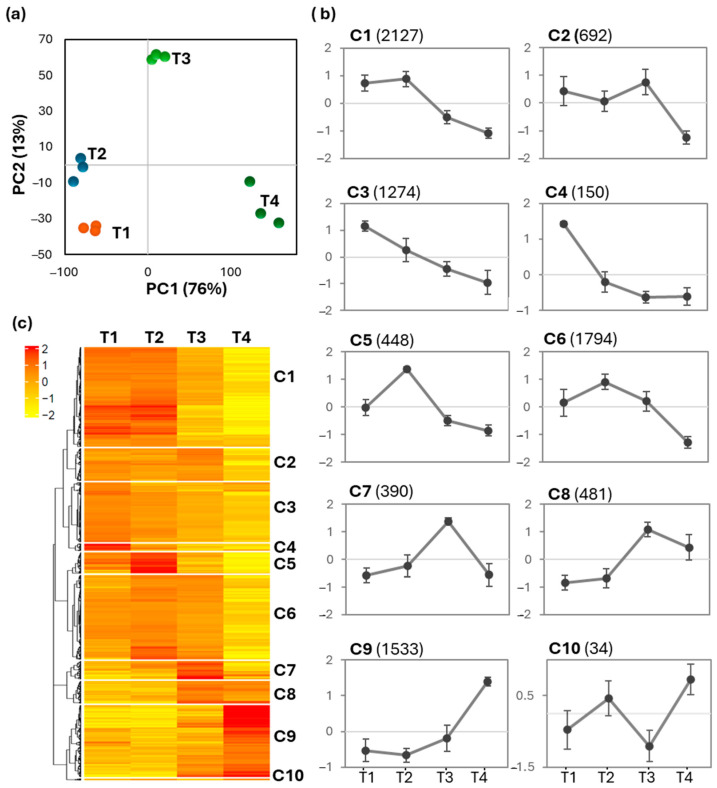
Transcriptomic profiles of cell suspension cultures growing in the bioreactors. (**a**) Principal component analysis of RNA-Seq data obtained from cells at the early-exponential (T1), late-exponential (T2), and stationary (T3) phase and three days after elicitation (T4). (**b**) Gene expression profiles of the ten clusters. The number of genes present in each cluster are indicated in brackets. The values are expressed as the rescaled RPKM values ± standard deviation. (**c**) Hierarchical clustering heat map of the RNA-Seq data. A Pearson correlation coefficient of >0.42 was used to define the ten clusters (C1–C10). The colors indicate expression intensity, with a scale bar provided for reference.

Principal component analysis (PCA) of the RNA-Seq data revealed that 89% of the variation is explained by the first (PC1, 76%) and second (PC2, 13%) principal components. The analysis clearly distinguished the different time points, indicating distinct transcriptomic signatures at each phase (Figure 4a). Specifically, T1 and T2 clustered together, separating from the other time points along PC1, which is predominantly associated with biological processes related to the cell cycle and cellular component organization. In contrast, T3 was clustered further away from the others along PC2, which consisted of pathways related to the translation process (Table 1). This clustering underscores the dynamic changes in gene expression that occur throughout the growth phases of the Cox cell suspension cultures.

Hierarchical clustering analysis of RNA-Seq data was conducted to define groups of co-regulated genes (Figure 4b). Ten clusters (C1–C10) with distinct expression profile were identified (Figure 4c and Supplementary Data S1). C1 includes 2127 contigs that exhibit higher expression level at T1 and T2 compared to the other two time points. C2 and C6 consist of 692 and 1794 contigs, respectively, both showing a significant decrease in expression upon elicitation (T4), while the ones of C3 (1274 contigs) and C4 (150 contigs) demonstrate a progressive decline over time. C5 and C6 comprise contigs (448 and 1794, respectively) with peak expression at T2, while C7 and C8 are represented by contigs (390 and 481, respectively) displaying the highest expression at T3. The elicitation-responsive genes are present in C9 (1533 contigs), exhibiting elevated expression levels at T4. Finally, C10 contains only 34 contigs, higher expression observed at both T2 and T4.

### 2.4. GOE Analysis

**Figure 5:**
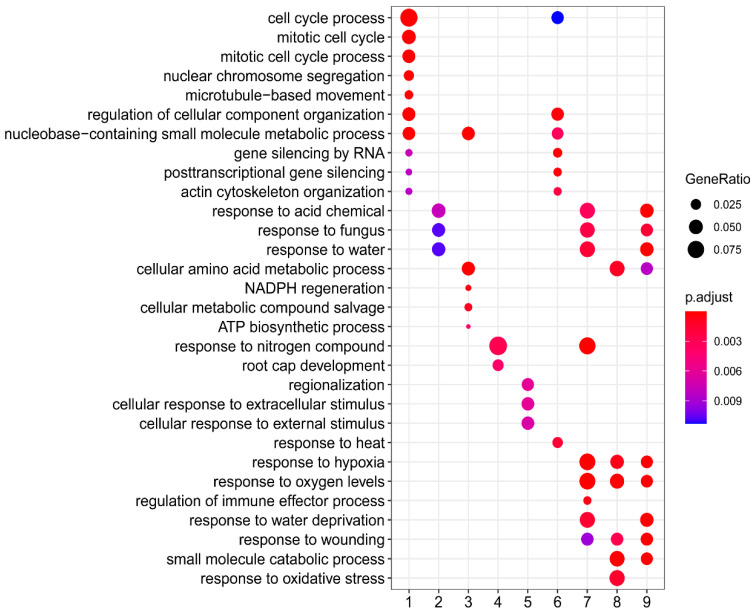
Gene ontology (GO) enrichment analysis of clusters. This figure displays the ten most significantly enriched biological processes at the GO level of 4 for each cluster, with the exception of C10, which did not show any enriched GO terms. The dots are color-coded based on the enrichment confidence (*p*-adjusted value), with darker colors indicating higher significance. Additionally, the size of each dot reflects the percentage of differentially expressed genes associated with the corresponding GO terms.

To understand the biological processes associated with each expression pattern, a gene ontology enrichment (GOE) analysis was performed for each cluster (Supplementary Data S1). The results, which summarize the ten most significantly enriched biological processes, are presented in Figure 5. Overall, the enriched GO terms varied among clusters; however, a notable similarity in GO enrichment patterns was observed across some clusters. For instance, both C1 and C6 contain gene sets associated with cell cycle-related processes and gene silencing. Additionally, GO terms related to the cellular response to stimuli (e.g., stress, water, fungus, hypoxia, etc.) were identified in C2, C7, and C9. This highlights the complex interplay of biological processes involved in the regulation of gene expression throughout the different phases of growth and in response to elicitation.

We hereafter first discussed the data concerning the general cellular activities of the cells in the suspension cultures across distinct phases, with a focus on processes associated with the cell cycle, cell wall, and lipids. To identify the biological pathways that are more specifically related to each growth phase, we further examined the ontologies enriched in C4, C5, C7/8, and C9, where contigs exhibited peak expression at T1 through T4, respectively (Figure 4c). This targeted analysis will help elucidate the specific molecular mechanisms and biological processes that characterize each growth phase and their roles in triterpene biosynthesis and overall cellular function.

### 2.5. Cell Cycle-Related Process and Its Regulation in Suspension Cell Cultures

The cell division cycle in eukaryotic cells consists of four sequential phases: G1 (postmitotic interphase), S (DNA synthesis phase), G2 (premitotic interphase), and M (mitosis) [40]. This cycle is intricately regulated by a variety of cellular processes that are temporally and spatially coordinated [40]. In this study, a full spectrum of cellular events characterizing the distinct phases of the cell cycle is highlighted in C1, where genes are upregulated at both T1 and T2. These events encompass key processes such as DNA replication, microtubule-based movement, microtubule/cytoskeleton organization, chromosome condensation, organization, separation, and nuclear division (Figure 5 and Supplementary Data S1). This finding correlates with the observed increase in sedimented volume of the cells from T1 to T2 (Figure 2A), indicating that the cells are actively dividing during this period. Thus, the upregulation of genes associated with cell division accurately reflects the cellular status during the exponential phase. Similar observations have been reported in studies involving poplar [37], thale cress [41], and tobacco BY2 suspension cells [22], reinforcing the notion that the transcriptional landscape during these phases is conserved across different plant systems.

Numerous studies have shown that the primary driving force behind cell cycle progression is the complexes formed between cyclin-dependent kinases (CDKs) and their cyclin partners (reviewed in [42]). Indeed, genes encoding plant-specific B-type CDKs, including CDKB1;1, CDKB1;2, and CDKB2;2, were exclusively identified in C1. It has been demonstrated that the *CDKB1;1* gene is activated at the onset of S-phase in both *A. thaliana* [43] and BY2 suspension cells [44], while *CDKB2;2* transcripts appear to be restricted to mitotic cells across various plant species [43,45,46]. Although we cannot directly associate the expression of these genes with specific cell cycle phases due to the lack of detailed assessment of the cellular status at each time point, our results highlight the presence of these plant-specific CDKs in Cox cells and their significant involvement in cell proliferation. Moreover, eight mitotic cyclin genes, including *CYCA1;1*, *CYCA3;4*, *CYCB1;2*, *CYCB1;4*, *CYCB2;3*, *CYCB2;4*, *CYCB3;1*, and *CYCD3;2*, are predominately present in C1 (Supplementary Data S1). This further supports the fundamental role of these cyclins in the cell cycle machinery by activating CDKs [42].

Some cell cycle-related processes are likely to remain active even during the stationary phase. This is evidenced by the enrichment of genes involved in DNA replication, mitotic cytokinesis and the regulation of chromosome and cytoskeleton organization in C6, where gene expression levels remained consistently high from T1 to T3 (Figure 4c and Figure 5, Supplementary Data S1). Among these genes, it is worth highlighting the presence of genes encoding cyclin-dependent kinase (CDKA;1) and small CDK-binding subunit 2 (CKS2). As one of the main cell cycle regulators of mitosis, *CDKA;1* has been shown to be constitutively expressed throughout the cell cycle in cell cultures of *A. thaliana* [43,47] and alfalfa [48]. Furthermore, studies on the cell cycle interactome in *A. thaliana* cell suspension cultures have revealed that CDKA;1 interacts sequentially with various cyclins, whose activities are regulated in a growth phase-dependent manner [47,49]. It is likely that CDKA;1 in Cox cell suspension cultures functions similarly, remaining constantly present while selectively forming complexes with cyclins. This suggests that CDKA;1 may participate in the regulatory network associated with specific phases of the cell cycle, thus facilitating cell cycle progression even during the stationary phase. This ongoing regulation underscores the complexity of cellular activities in suspension cultures and highlights the importance of CDKs in maintaining cellular functions throughout different growth stages.

Other features of cell cycle control are also evident during the exponential growth phase of Cox cells. For example, *E2F*s, transcriptional factors that promote DNA replication [40], were clustered in C1, C3, and C5, showing elevated expression during early- and/or late-exponential phases (T1 and/or T2). Moreover, genes encoding CDKs inhibitors (KRP3 and KRP7), exhibited peak expression at T2, which may regulate cell cycle progression by binding to S-phase cyclin-CDKs, as previously reported [50,51]. Furthermore, genes encoding the core components of the spindle assembly checkpoint (SAC), such as BUB1, BUB3.1, MAD1, MAD2, and BUBR1, were also identified in C1. This indicates that the fidelity of chromosome segregation during Cox cell mitosis is likely governed by the SAC, similar to observations in thale cress, yeast, and animal systems [52,53].

In addition to the DNA replication process, GO terms associated with DNA damage response and repair were also found in C1 and C6 (Supplementary Data S1). On the one hand, enhanced DNA repair and correction processes are essential for proliferating cells to ensure normal cellular function and maintain genetic stability [54]. On the other hand, this can also be partially attributed to environmental stresses typically present in bioreactors, such as oxygen deprivation, shear stress, and fluctuations in pH [55,56]. Supporting this, there was a concomitant enrichment of GO terms related to the cellular response to stress in both clusters (Supplementary Data S1), as well as observed reductions in dissolved oxygen levels and oscillations in pH during the production process (Figure 2B,C). Studies have shown that a limited oxygen level leads to a lower growth rate and delays in sugar uptake during the proliferation phase of carrot cell cultures [57]. The authors also noted enhanced alcohol dehydrogenase (ADH) activity under hypoxic conditions, suggesting an induced alcoholic fermentation response to cope with reduced oxygen concentration. Interestingly, *ADH* was found in C2 and C6, maintaining a consistently high expression level from T1 to T3. This result indicates that a similar adaptive mechanism may be triggered by elevated demand for oxygen levels in Cox cell suspension cultures, highlighting the interplay between environmental factors and cellular responses during the growth phases.

Moreover, the activation of the DNA integrity checkpoint in response to DNA damage is also indicated by the presence of the *WEE1* gene in C1. *WEE1* is transcriptionally activated upon DNA damage and is recognized as a critical target for DNA replication checkpoints; it plays a prominent role in cell cycle arrest, permitting cells to repair damaged DNA before proceeding to mitosis [58].

Taken together, the data presented herein suggest that the cell cycle-related processes in Cox cell suspension cultures likely share common characteristics with those observed in other studied plant species. Additionally, cells growing in bioreactors are probably exposed to environmental stresses that could impede normal cell proliferation. Therefore, there is potential for further improvement in biomass yield and growth rates by fine-tuning the operating parameters of the bioreactor to minimize stress. In the present setup, to better understand the physiology of our cell model, we did not implement any regulation of the dissolved oxygen level through airflow or stirring speed cascades. However, strategies to maintain sufficient dissolved oxygen concentrations should, of course, be implemented during production processes. In addition to this, other parameters such as temperature, pH, or shear forces should be fine-tuned to further optimize cell growth conditions. We here provide a set of candidate genes that could serve as useful markers for monitoring cell behavior, which, in combination with molecular biology approaches, could help optimize the cell division rate in plant cell culture processes.

### 2.6. Cell Wall-Related Process

De novo synthesis of the cell wall plays a vital role in partitioning the cytoplasm between daughter cells during cytokinesis, which is crucial for the final stages of plant cell division [59,60]. It is widely accepted that cell wall synthesis begins with the assembly of the cell plate, a process that involves the delivery and fusion of Golgi-derived vesicles carrying cell wall and cell membrane components [61]. During this process, callose is formed as a major luminal component of the cell plate/nascent cross-wall [62,63]. Mature cross-walls are composed similarly to primary cell walls, primarily consisting of cellulose, xyloglucans, and pectins [64]. In this study, genes associated with cell wall biosynthesis were predominantly enriched in C1, corresponding to the exponential growth phase (Supplementary Data S1). We will hereafter discuss the results regarding the biosynthesis of key cell wall components.

1. Callose

Direct evidence for callose deposition at cell plates was provided by [62], who immunolabeled sections of dividing tobacco BY2 cells and root tips using an antibody against callose. They observed the transient accumulation of callose during various phases of the cell plate assembly, as well as in parental cell walls at sites connecting to the nascent cross-wall. Callose was thought to provide mechanical strength and rapid spreading force to the tubular membrane network, facilitating cell plate formation until the load-bearing cellulose network is established. Additionally, the necessity of callose for connecting the nascent cross-wall to the parental wall was highlighted in the study of *A. thaliana* mutants deficient in callose synthase 8 (GSL8), which exhibited cytokinesis-defective phenotypes [65]. This notion is reinforced by our data showing peak expression of *GSL8* during the late-exponential phase (C6, Supplementary Data S1). Other members of the GSL family were also identified in this study (e.g., GSL12 in C1 and 2, *GSL3* in C2, and *GSL3*, *5*, and *12* in C6), indicating the potential involvement of these genes in the cell cycle process. Taken together, these findings suggest that callose deposition is closely linked to cytokinesis and new cell wall synthesis in dividing Cox cells.

2. Cellulose

Cellulose is the primary load-bearing component of the primary cell wall and is synthesized by plasma membrane-localized cellulose synthase complexes (CSCs). These complexes most likely consist of cellulose synthase (CESA) proteins organized in hexameric rosettes, including CESA1, 3, 6, and 6-like proteins in primary cell walls [66,67,68]. Miart et al. [69] proposed that cellulose synthesis plays an essential role during the early stages of cell plate assembly, based on their study of GFP-labeled CESAs and observations of cellulose deposition in developing cell plates using fluorescence imaging. This assertion is supported by phenotypic studies of *A. thaliana cesa1* mutants, which exhibit aborted cell plates [70]. Furthermore, the exogenous application of a CSC inhibitor (C17) in *A. thaliana* cell suspension cultures led to impaired cytokinesis, further supporting the interdependent relationship between cell division and cellulose synthesis [71]. In this study, we observed *CESA1*, *CESA3*, and *CESA6* in both C2 and C6, indicating high expression levels from T1 to T3 (Supplementary Data S1). This suggests that the same rosette CSC may be responsible for cellulose synthesis during both cytokinesis and cell expansion in Cox suspension cells.

3. Pectin

Ontologies associated with the pectin biosynthetic process were identified in C1, suggesting that pectic polysaccharides are synthesized during the exponential growth of Cox cells. Among the genes enriched in this category, six out of nine belong to galacturonosyltransferase (GAUT) and GAUT-like (GATL) gene families, including *GAUT4*, *6*, *8*, *9*, *10,* and *GATL3*. Mutants of *A. thaliana* deficient in each of these genes, except for *gaut4*, exhibited reduced levels of galacturonic acids in their cell walls. The failure to obtain homozygous progeny from *gaut4* mutants further underscores its essential role [72]. Based on its high expression and amino acid similarity to GAUT1, which is known to participate in homogalacturonan (HG) synthesis, GAUT4 is also proposed to be involved in HG synthesis [72]. Moreover, *TUMOROUS SHOOT DEVELOPMENT2* (*TSD2*, as known as *QUA2*, MD00G1010000) encoding a pectin methyltransferase, was also observed in C1 along with the *GAUTs* [73]. This suggests that methyl-esterified HG is synthesized in the Cox cells during cell wall construction. Similar results have been reported for both tobacco BY2 and thale cress dividing cells, where methyl-esterified HG was detected at the cell plate as shown by immunolabelling with the JIM7 antibody [74,75].

It is worth mentioning that genes involved in the biosynthesis of rhamnogalacturonan II (RGII), another pectic polymer, were upregulated during the exponential growth of Cox cells. These include orthologs of 3-deoxy-D-manno-octulosonic acid-8-phosphate (Kdo-8-P) synthase 1 (*ATKDSA1*, MD10G1213400) in C1, and male gametophyte defective 2 (*MGP2*), also known as sialyltransferase-like 1, (MD09G1003400 and MD17G1007200), in C6 (Supplementary Data S1). RGII is a highly complex polysaccharide with side chains containing rare and thus diagnostic sugars such as Kdo. Its phosphorylated precursor is synthesized by Kdo-8-P synthases (KDSAs) [76,77,78]. The absence of Kdo biosynthesis has been shown to impair pollen tube elongation, with ATKDSA1 contributing to 36% of the total cytosolic Kdo in thale cress [79].

Interestingly, Delmas et al. demonstrated that *KDSA* expression and enzyme activity are linked to cell proliferation, especially in meristematic tissues and young tomato fruits [80]. In tobacco BY2 cells, *KDSA* expression was found to oscillate during the cell cycle, peaking in the M phase. The upregulation of *ATKDSA1* during the exponential growth of Cox cells may similarly contribute to Kdo accumulation and, thus, RGII synthesis during the de novo cell wall formation in dividing cells. This hypothesis is further supported by the concomitant upregulation of *MGP2*, which is thought to transfer Kdo onto the HG backbone of RGII. The *mgp2* mutant of *A. thaliana* exhibited impaired pollen germination and pollen tube growth, reinforcing the potential role of MGP2 in RGII synthesis [81].

4. Hemicelluloses

Xyloglucan (XyG) is a crucial hemicellulose component in the primary cell walls of non-graminaceous plants, where it tightly binds to cellulose microfibrils to form the dominant tension-bearing network, supporting the growing cell wall structure [82]. In this study, the cellulose-synthase-like C4 gene (*CSLC4*) was significantly upregulated during the exponential growth phase (clustered in C1; T1 and T2 versus T3 > 3.7 and 2.5, receptively). This upregulation indicates that the synthesis of XyG backbone plays a key role in the cell division of Cox cells [83]. This observation is consistent with the findings of Moore et al. [84], who showed the presence of XyG in the forming cell plate and Golgi stacks during cytokinesis, using immunoelectron microscopy. These findings collectively suggest that XyG is actively synthesized during cell division to form the essential structural components of the cell wall.

The importance of xyloglucan endotransglucosylase/hydrolase (XTH) enzymes in cell expansion has been well documented, primarily due to their ability to cleave and/or rejoin xyloglucan chains, thereby modulating cell wall extensibility [85]. The expression and activity of XTH have been closely linked to growth [86]. In fact, exogenous applications of XTH [87] and modulation of *XTH* expression [88,89,90] have demonstrated effects on growth and cell wall mechanics.

In the present study, several *XTH,* including *XTH6*, *15*, *16* and *23,* were found in C7, with peak expression during the stationary phase (T3). This timing suggests that their role in the Cox cells may primarily relate to cell expansion after the completion of cell division. This result aligns with the observed increase in cell size as the culture approached the stationary phase (Figure 1b), further supporting the role of XTHs in modifying the cell wall to accommodate cell enlargement at later growth stages.

5. Expansins

Expansins are crucial proteins that facilitate cell enlargement by loosening the cell wall matrix, enabling turgor-driven expansion through nonenzymatic disruption of noncovalent bonds within the wall polysaccharide network [91,92]. This loosening effect allows for the rearrangement of cellulose–hemicellulose networks, aiding in cell wall extensibility and expansion. In earlier research, Link et al. demonstrated that expansins are naturally present in tobacco BY2 suspension cells at sub-saturating levels, and exogenously applied expansins were shown to induce cell enlargement [93]. In this study, eight contigs coding for expansin (EXP) orthologs were identified. Of these, three were found in C1, with higher expression levels observed during both early- and late-exponential phases, specifically *EXPA1*, *EXPA13*, and *EXPA20*. Another four expansin genes, including *EXPA4*, *EXPA13*, and *EXPB3*, were observed in C5 with peak expression at T2, and one expansin-like gene (EXP-like A2) was identified in C2 with a consistently high expression from T1 to T3 (Supplementary Data S1). These findings suggest that EXPs may have overlapping yet distinct roles across different developmental stages of Cox suspension cells. Although cell enlargement typically occurs during the stationary phase, as discussed above, the high levels of *EXP*s expression at earlier time points (T1 and T2) suggest that EXPs might also play a role in cell division. For instance, EXPA1 was previously shown to regulate lateral root initiation by controlling asymmetric pericycle cell divisions in *A. thaliana* [94]. Future studies examining protein abundance at various time points would provide further insights into the precise roles of EXPs in the growth and development of Cox suspension cells.

### 2.7. Lipids

Lipid metabolism plays a critical role during cell division, organelle formation, and responses to environmental stress in plant cells. In Cox suspension cells, genes involved in lipid, sterol, and fatty acid biosynthesis were predominantly clustered in C1, indicating a high demand for membrane and organelle construction during exponential growth. In particular, the biosynthesis of polyunsaturated fatty acids (PUFAs), especially linolenic acid (18:3), was highlighted in C5 at T2. Key genes, such as fatty acid biosynthesis 1 (*FAB1*), fatty acid desaturase 3 (*FAD3*) and *FAD7* exhibited peak expression levels at this phase (Supplementary Data S1).

PUFAs play important roles in enhancing membrane fluidity and participating in stress signaling, making them crucial during stress responses [95,96,97]. Among various stressors, phosphate deprivation is particularly known to trigger a halt in cell division and initiate membrane lipid remodeling in plant cell cultures [98]. In this study, the gene UDP-glycosyltransferase (*SQD2*), is notable for its role in lipid remodeling during phosphate starvation [99]. Taken together, cells at the T3 are likely to promote PUFAs synthesis as part of stress response to the deprivation of nutrition in the culture media.

By T3, there was a marked increase in the expression of genes associated with the lipid catabolism (clustered in C8). This suggests that lipid turnover, possibly indicative of cellular senescence, may be occurring in response to severe nutrition depletion. Lipid catabolism, including the breakdown of fatty acids through beta-oxidation, provides signaling molecules, adjusts membrane composition, and generates metabolic intermediates [6]. The observation of fatty acid beta-oxidation in this cluster aligns with the notion that nutrient stress at this stage triggers the breakdown of lipids to sustain energy production and maintain cellular homeostasis [100].

### 2.8. Biological Processes Related to Early-Exponential Phase

To investigate the biological processes specifically associated with the early-exponential phase (T1) in Cox suspension cells, we examined gene ontology (GO) terms enriched in C4 (Supplementary Data S1), which is characterized by genes with peak expression at this stage. One notable finding is the enrichment of genes involved in nitrate transport, indicating the importance of nitrate uptake during early cell growth and division. This is likely linked to the need for de novo protein synthesis, a key requirement during cell proliferation. Nitrate uptake is known to be an inducible process, as shown by studies in barley roots, where nitrate or nitrite presence in the medium triggers protein synthesis for effective nitrate absorption [101]. Depending on external nitrate concentration, plants switch between low- and high-affinity nitrate transport systems [102,103], and interestingly, genes from both systems were identified in this study: nitrate transporter (*NRT*)*1.5* and *NRT1.9* (low-affinity) and *NRT2.5* (high-affinity) (Supplementary Data S1). This suggests that cells adapt nitrate uptake mechanisms based on nutrient availability in the culture medium. Furthermore, the upregulation of genes involved in ammonium uptake during the exponential phase of cell growth was observed. Specifically, genes such as ammonium transporter 1 (*AMT1*), clustered in C1 and C4, and *AMT2*, clustered in C6, show increased expression during this phase. Following inoculation, a significant pH drop from 4.9 to 3.7 in the first two days was recorded. This pH decrease can be explained by the cells’ preference for ammonium uptake, as ammonium assimilation releases protons (H^+^) into the culture medium, thereby acidifying it. As cell growth continues, a shift from ammonium uptake to nitrate uptake likely occurs. This transition is typically associated with an increase in pH because nitrate assimilation involves the consumption of protons, resulting in a more alkaline environment. This dynamic change in nitrogen source utilization reflects the cells’ ability to adjust their nutrient uptake mechanisms based on the availability of ammonium and nitrate in the medium, optimizing their growth as conditions change.

GO terms related to response to chitin and flavonoid biosynthetic processes were enriched, which could reflect cellular defense mechanisms in response to environmental stress [104]. Flavonoids are known to enhance plant tolerance to various stressors [105], and their biosynthesis may be activated here as part of an early stress response. Although Cox cells were grown in aseptic conditions, the transition from shake flasks to bioreactors introduced new physical and nutritional challenges. These changes, including variations in stirrer speed, aeration, and nutrient levels, might trigger stress responses as cells adapt to the bioreactor environment.

Another notable finding in C4 is the enrichment of genes involved in the toxin catabolic process, particularly glutathione transferase (*GST*) genes. GSTs play a pivotal role in detoxifying toxins through glutathione conjugation [106]. Their upregulation during the early-exponential phase suggests that cells were actively detoxifying harmful by-products, such as reactive oxygen species (ROS), which are commonly produced during high metabolic activity. This detoxification process is essential to protect cells from oxidative damage and ensure the continuation of normal cellular functions, particularly during rapid growth when the risk of oxidative stress is elevated. By neutralizing these toxic compounds, GSTs help maintain cellular homeostasis and support healthy proliferation.

### 2.9. Biological Processes Related to Late-Exponential Phase

During the late-exponential phase of Cox cell growth, genes with peak expression at T2 were clustered in C5. GO analysis indicates that Cox cells are actively responding to extracellular stimuli, such as auxin and nutrient levels, during this phase. Specifically, pathways related to auxin polar transport and the auxin-activated signaling pathway were highlighted, underscoring the role of auxin, a critical phytohormone, in regulating various aspects of plant growth and development, including cell differentiation.

Another key finding during this phase is the over-representation of genes involved in the cellular response to phosphate starvation. One such gene is phosphate starvation-induced gene 2 (*PS2*), a well-established marker of phosphate deficiency in plants like *Arabidopsis thaliana*. PS2 plays a critical role in maintaining intracellular phosphate homeostasis during phosphate scarcity by immediately cleaving pyrophosphate [107]. This suggests a high demand of phosphate during the growth of Cox cells, as phosphate is essential for synthesizing various cellular components, such as lipids (as described above), nucleic acids, ATP, etc. Given that phosphate deficiency is likely a limiting factor in the growth of Cox cells, it may impair cell division, as reported in other plant cell suspension cultures, including *tobacco* [98] and *Catharanthus roseus* [108]. The identification of phosphate starvation responses implies that optimizing phosphate levels in the culture medium, either by increasing initial concentrations or through re-supplementation during cell growth, could enhance biomass yield in Cox cell suspension cultures.

### 2.10. Biological Processes Related to Stationary Phase

During the stationary phase of Cox cell cultures, significant biological changes occurred as cells adapted to both external and internal stressors. Although cell growth ceased, the cells remained highly responsive to various external stimuli, such as cytokinin, salicylic acid, jasmonic acid, light intensity, oxygen, water, and nutrients (as reflected in C7 and C8). This is consistent with prior research on tobacco BY2 cells, which also demonstrated that cells in the stationary phase maintain responsiveness to phytohormones and extracellular signals [22].

Abiotic and biotic stress factors, such as hypoxia, water deprivation, oxidative stress, nutrient starvation, and even potential exposure to bacterial or viral agents, are likely to be prevalent at this stage. For instance, dissolved oxygen levels in the culture medium dropped to approximately 10% (Figure 2), which coincides with the peak expression of genes related to the cellular response to decreased oxygen levels. This hypoxic condition might have triggered a cascade of defense mechanisms as cells attempt to survive and maintain homeostasis. Furthermore, as previously discussed, cell expansion was observed during this phase, likely contributing to stress due to increased turgor pressure driving cell enlargement. While elevated turgor pressure is essential for cell growth and expansion, excessive pressure can lead to cellular stress if it surpasses the cell’s structural limits [5]. Additionally, larger, expanded cells are more prone to physical contact, which can increase friction between cells and potentially exacerbate mechanical stress.

To manage these stressors, cells activated different defense pathways. Notably, autophagy was initiated to remove damaged cells and maintain cellular function. This self-digestion process was supported by the activation of catabolic pathways that break down cellular components such as lipids, organic acids, and amino acids. Moreover, the upregulation of genes related to trehalose biosynthesis in C8 suggests the accumulation of trehalose, a known osmoprotectant that helps cells cope with osmotic stress.

Maintaining cellular ion homeostasis becomes crucial at this stage, as reflected by the activation of ion transmembrane transport and its regulation in C8. Cells likely activated nutrient uptake mechanisms, particularly for amino acids and carboxylic acids, to compensate for nutrient deficiencies, with transporter genes peaking in expression (C7). This indicates that nutrient acquisition from the extracellular environment is essential for survival as resources become limited during the stationary phase.

### 2.11. Biological Processes Induced upon Elicitation

Upon elicitation with MeJA in Cox cell suspension cultures, a broad range of biological processes related to cellular responses to both endogenous and exogenous stimuli, as well as defense responses to various stresses, were activated (C9, Supplementary Data S1). This is consistent with the established role of MeJA as a key signaling molecule in plant defense, particularly in response to wounding and pathogen attacks.

Previous research has shown that MeJA application can enhance the production of secondary metabolites, including phenylpropanoids, terpenoids, alkaloids, and flavonoids, in various plant species and cell suspension culture (reviewed in [109]). In this study, we observed the upregulation of genes related to the general phenylpropanoid biosynthetic process, such as those encoding phenylalanine ammonia-lyase 1 (PAL1, MD01G1106900) and 4-coumarate:CoA ligase 3 (4CL3, MD07G1309000). These genes are involved in the production of *p*-coumaroyl-CoA, a precursor for flavonoid biosynthesis, suggesting an enhanced flavonoid production after MeJA elicitation. This is further supported by elevated expression levels of genes participating in flavonoid biosynthesis. This included four genes (MD15G1131700, MD15G1132000, MD15G1132100, and MD15G1132200) encoding chalcone synthase (CHS), which catalyzes the first committed step in flavonoid biosynthesis [110]; a gene encoding flavonol synthase (FLS, MD01G1153600); as well as genes encoding dihydroflavonol 4-reductase (DFR, MD11G1229100) and UDP-glucose: flavonoid-3-O-glucosyltransferase 3 (UFGT, MD09G1141700, and MD09G1141200), both of which play a role in anthocyanin biosynthesis [110]. These findings differ from those of [33], who reported no upregulation of key entry-point genes into the phenylpropanoid and flavonoid pathways, such as PAL and CHS, in *Medicago truncatula* cell cultures treated with MeJA. This contrast highlights the species-specific and context-dependent nature of MeJA-mediated secondary metabolite biosynthesis.

### 2.12. Transcriptional Profile Related to Triterpene Biosynthesis

**Figure 6:**
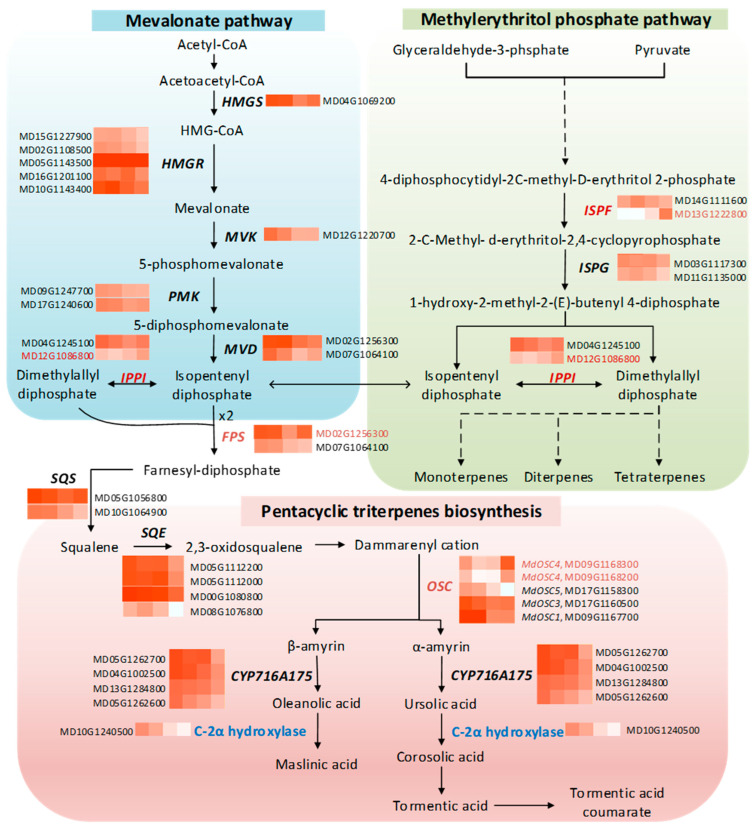
Changes in the expression level of key genes involved in the mevalonate, methylerythritol phosphate, and pentacyclic triterpene biosynthesis pathways in Cox cell suspension cultures. Colored boxes represent the normalized intensity of transcript levels across different time points: early-exponential, exponential, and stationary phase and 2 days after elicitation. HMGS, 3-hydroxy-3-methylglutaryl-CoA synthase; HMGR, 3-hydroxy-3-methylglutaryl-CoA reductase; MVK, mevalonate kinase; PMK, phosphomevalonate kinase; MVD, mevalonate5-diphosphate decarboxylase; IPPI, isopentenyl diphosphate isomerase; FPS, farnesyl pyrophosphate synthase; ISPF, 2-C-methyl-d-erythritol 2,4-cyclodiphosphate synthase; ISPG, 4-hydroxy-3-methyl but-2-enyl diphosphate synthase; SQS, squalene synthase; SQE, squalene epoxidase; CPY716A175, C-28 oxidase; OSC, oxidosqualene cyclase. Solid and dashed arrows denote single and multiple enzymatic steps, respectively. The upregulated genes upon elicitation are highlighted in red. The putative gene is indicated in blue.

No GO term related to triterpene biosynthesis was significantly enriched in our study. Therefore, we closely examined the expression changes in key genes involved in triterpene biosynthesis across various time points. Triterpenes are produced from universal precursors: isopentenyl diphosphate (IPP) and its isomer dimethylallyl diphosphate (DMAPP). These precursors are derived from the methylerythritol phosphate (MEP) pathway in the plastids and the mevalonate (MVA) pathway in the cytoplasm. Previous studies have shown that exogenous application of MeJA enhances the expression of genes in both pathways in cell suspension cultures of various plant species, such as *Panax ginseng* [36] and *Andrographis paniculate* [111]. In contrast, our study found that genes related to the MVA pathway were predominantly clustered in C1 and C3, showing decreased expression at both the stationary phase (T3) and three days post-elicitation (T4). These genes include 3-hydroxy-3-methylglutaryl-CoA synthase (*HMGS*), 3-hydroxy-3-methyl-glutaryl-CoA reductase (*HMGR*), mevalonate kinase (*MK*), phosphomevalonate kinase (*PMK*) and diphosphomevalonate decarboxylase (*MVD*) (Figure 6, Supplementary Data S1). Notably, two out of five HMGR-encoding contigs (MD05G1143500 and MD16G1201100) exhibited a marked decrease in transcript levels under MeJA treatment (clustered in C2).

In contrast, one contig (MD12G1086800) encoding isopentenyl diphosphate isomerase (IPPI) was found to be upregulated following MeJA treatment. IPPI catalyzes the interconversion of IPP and DMAPP, a critical step for balancing building blocks needed for desired terpenoids biosynthesis. IPPI features in both the MEP and MVA pathways and it is indispensable in the latter for producing DMAPP in the cytosol [112]. Overexpression of *IPPI* in the cytosol of *Artemisia annua* increased artemisinin content by boosting DMAPP levels available for artemisinin biosynthesis [113]. The same study also demonstrated the transport of additional IPP from plastids to the cytosol in transgenic plants. Similarly, in our study, the peak expression of *IPPI* upon MeJA application suggests an enhanced conversion of IPP to DMAPP in the cytosol. Therefore, the increased triterpene content observed after elicitation might be partially due to the higher availability of the building blocks. This is further supported by the elevated expression of farnesyl pyrophosphate synthase (*FPS*, MD02G1256300), which catalyzes the condensation DMAPP and IPP to produce farnesyl diphosphate, a precursor for short-chain terpenoids (Figure 6).

The possible transport of additional IPP from plastids (via the MEP pathway) to the cytosol is implied by the downregulation of the MVA pathway, concurrent with the upregulation of *2-C-methyl-d-erythritol 2,4-cyclodiphosphate synthase* (*ISPF*, MD13G1222800), a key MEP pathway gene (Figure 5).

Most genes involved in the triterpene biosynthesis, such as squalene synthase (*SQS*)*,* squalene epoxidase (*SQE*), C-28 oxidase (*CPY716A175*), were more expressed during the early- and late-exponential phase, suggesting that the triterpene biosynthesis pathway is an important component during the cell division. Previous studies showed that pentacyclic triterpenes play a structural role of plant surface waxes and cell wall [114,115]. Given this, we might speculate that, in Cox cells, pentacyclic triterpenes are synthesized concomitantly with the formation of membranes and the cell wall during cell division. Conversely, two contigs encoding oxidosqualene cyclase (OSC, MD09G1168300 and MD09G1168200) were strongly upregulated by MeJA, with expression levels that increased approximately 20-fold compared to T3 (Figure 5, Supplementary Data S1). Sequence comparison with different apple *OSC*s (*MdOSC*s) identified these two contigs as *MdOSC4.* Previous studies have shown that the transient expression of *MdOSC4* in *Nicotiana benthamiana* leaves results in the production of oleanane-type triterpene (putative germanicol), β-amyrin and lupeol at a ratio of 82:14:4; however, germanicol was not detected in the skins of any of the 20 apple cultivars studied [116]. The authors hypothesized that germanicol may be rapidly converted into unknown metabolites or/and quickly catabolized for fruit growth and development. In our studies, we observed a significant increase in maslinic acid (an oleanane-type triterpene) after elicitation; however, we could not explain the significant increase in ursane-type triterpenes, including tormentic acid, corosolic acid, and tormentic acid coumarate, based on our data. We hypothesize that some form of post-transcriptional regulation may be occurring, which could affect the synthesis of these compounds without a corresponding change in gene expression levels. Additionally, it is possible that the role of MdOSC4 might not be fully understood, particularly given the multifunctional role of OSCs [116]. In-depth gene-functional characterization in Cox cell suspension cultures, along with comprehensive triterpene profiling, will provide more light into whether or how *MdOSC4* contributes to triterpene biosynthesis.

The predominant triterpenes in Cox cell suspension cultures include ursane-type (corosolic acid and its derivatives: tormentic acid and coumaroyl-tormentic acid) and oleanane-type (maslinic acid) (Figure 3). The synthesis of these triterpenes requires an enzyme that catalyzes the C-2α hydroxylation of ursolic acid and oleanolic acid. A C-2α hydroxylase, CYP716C55, has been identified in the medicinal tree banaba [117]. BLASTX analyses against the transcriptome of Golden Delicious, using the sequence of *CYP716C55,* identified a highly similar sequence with 77.54% identity (accession number MD10G1240500; *E*-value: 0). This gene, present in C4, exhibited a declining expression across time points, and could be a putative *C-2α hydroxylase* responsible for the C-2α hydroxylation of ursolic and oleanolic acids in Cox cells. Further gene-functional studies and detailed triterpene profiling will clarify the role of *MdOSC4* and other enzymes in triterpene biosynthesis.

## 3. Materials and Methods

### 3.1. Callus Induction, Cell Suspension Culture, and Cell Suspension Maintenance

Calli of the apple (*Malus* × *domestica*) cultivar “Cox Orange Pippin” were initiated from fruit flesh parenchymatic explants at 23 °C in the dark. Apple fruits were peeled, sterilized by soaking in 5% commercial bleach solution for 15 min, and then washed three times with sterile distilled water. The fruits were then cut into pieces approximately 0.5 mm thick and placed on solid Linsmaier and Skoog (LS) medium [118] with a pH of 5.8. This medium was supplemented with 30 g/L sucrose, 1 mg/L 2,4-Dichlorophenoxyacetic acid (2,4-D), 0.5 mg/L kinetin and 8 g/L agar (Kalys, Bernin, France). Calli were subcultured monthly on the calli-maintaining medium, which was solid LS medium containing 30 g/L sucrose, 0.2 mg/L 2,4-D and 0.2 mg/L 1-Naphthaleneacetic acid (NAA).

Cell suspension cultures were established by resuspending calli in the same calli-maintaining medium without agar. These cultures were maintained in an Innova 44R incubator (Eppendorf, Hamburg, Germany) at 22 °C and 120 rpm in the dark. Subcultures were performed at three-week intervals with 20% (*v*/*v*) inoculum.

The callus culture was initiated in 2014 and has been regularly subcultured every four weeks. The cell suspension cultures were established in 2016 with subculturing at two-week intervals. The experiments in shake flasks and bioreactors were conducted in 2018 and 2019, respectively. Prior to this bioreactor experiment, the cell cultures were routinely cultivated in bioreactors for other studies. Thus, both the callus and cell cultures have undergone sufficient passages to ensure stability, minimizing the risk of variability.

### 3.2. Growth Kinetics in Flasks

The growth kinetics of the cell suspension culture was assessed by measuring cell densities using a cell growth quantifier (CGQ, Scientific bioprocessing). For this purpose, six shake flasks, each containing 100 mL of cell suspension culture with a 20% (*v*/*v*) inoculum, were used. Measurements were taken every 2 days until 22 days after inoculation.

### 3.3. Bioreactor Operations and Elicitation

Three Minifors 2 bioreactors (Infors AG, Bottmingen, Switzerland) with 6 L vessel were used for the experiment, each with a working volume of 4 L. Calibration of the pH and dissolved oxygen (DO) probes (Hamilton Bonaduz AG, Bonaduz, Switzerland) were performed before and after sterilization, respectively. The dissolved oxygen probe was calibrated using a two-point calibration at 0% and 100% oxygen saturation of the media with N_2_ and air, respectively. Two 5.5 cm diameter elephant ear-type impellers were positioned at 0 cm and 12 cm above the bottom of the stirring bar. Three bioreactors were configured identically.

Once the temperature stabilized at 22 °C with an airflow of 0.5 *v*.*v*.*m* and an agitation speed of 150 rpm, the bioreactor was inoculated with 15% (*v*/*v*) of a 1-week-old cell suspension culture in the exponential phase, resulting in a final culture volume of 4 L. Growth parameters were monitored and recorded using EVE software (version 2023 H2, Infors AG, Switzerland).

To estimate the growth of the cell suspension culture, sedimented cell volume (SCV) was measured every 2–3 days, following a modified version of the method described by Blom et al. [119]. Briefly, stirring and airflow were paused for 6 min to allow the cells to settle, and the SCV was measured along the vessel wall using a ruler. The value was then converted into liters to obtain the SCV measurement.

MeJA (Merck, Darmstadt, Germany) was added to the bioreactors 25 days after inoculation following culture sampling (see below), to achieve a final concentration of 100 µM. A 50 mL culture was collected from each bioreactor using a sterile sampler at four time points: T1 = day 13, T2 = day 20, T3 = day 25, and T4 = day 28. The cells were vacuum-filtered and immediately flash-frozen in liquid nitrogen for subsequent analysis.

At the end of the bioreactor run, the remaining cell suspensions were collected and filtered through a 25 µm Miracloth using a Büchner funnel, and the fresh weight of the cells was measured. The cells were then stored at −80 °C. After one day, the frozen cells were transferred into a freeze dryer (Christ-Alpha 2–4 LSC plus, Martin Christ, Osterode am Harz, Germany), where they were lyophilized until their weight stabilized. Once the cells reached a constant weight, the dry weight was recorded.

### 3.4. Triterpenes Extraction and Analysis

The “Cox Orange Pippin” apple fruits were collected, and the skin was peeled as described by [17] and immediately frozen in liquid nitrogen. Briefly, the skin samples were generated from apples collected from three individual trees, representing three biological replicates, with each replicate consisting of a pool of 10 apples. “Cox Orange Pippin” cells were directly frozen in liquid nitrogen after filtration. The samples were freeze-dried for 48 h and ground using a Retsch ZM200 mill equipped with a 120 µm sieve (Retsch, Haan, Germany). Twenty-five milliliters of absolute ethanol was added to 100 mg of freeze-dried material. The mixture was agitated at 150 rpm for 1 h at room temperature. The extracts were further recovered through consecutive filtration using 45 µm (glass fiber) and 0.22 µm (PTFE ww) filters. Three biological replicates were used for the analysis. The separation and detection of the triterpenes were performed using Ultra-High-Performance Liquid Chromatography with Diode-Array Detection (UHPLC-DAD, Acquity U-HPLC-DAD, Waters, Milford, MA, USA), following the method previously described by Andre et al. [26]. The analysis utilized an Acquity UPLC BEH C18 column (2.1 × 100 mm, 1.7 µm) at 40 °C. The mobile phase consisted of methanol (solvent B) and water with 0.05% phosphoric acid (solvent A). Triterpenes were eluted at a flow rate of 300 µL/min. The gradient program began with 25% solvent A for 2 min, decreased to 18% solvent A until 16 min, further decreased to 0% solvent A until 25 min, and remained at 0% solvent A for an additional 1.5 min. Triterpenes were identified based on their retention times and spectral data, compared with certified standards, and quantified at 200 nm using external calibration.

### 3.5. RNA Extraction

Fresh cell samples were ground to a fine powder using a mortar and pestle in liquid nitrogen. Due to the high water content of the cells (approximately 95%), we increased the starting material mass to obtain a sufficient amount of total RNA. Total RNA was extracted from 1 g of cells using the RNeasy Plant Mini Kit (QIAGEN, Leusden, The Netherlands) with an adjusted protocol. The volume of RLT buffer was modified according to the starting material quantity as per the manufacturer’s guidelines. The lysis buffer was applied to the RNeasy column using a 3-pass method. The remaining steps, including an on-column DNase I treatment (QIAGEN, Leusden, The Netherlands), were performed following the manufacturer’s instructions.

Total RNA integrity was evaluated using the RNA Nano 6000 assay (Agilent Technologies, Diegem, Belgium) and a 2100 Bioanalyzer, with quality parameters tailored for plant RNA profiles (Agilent Technologies, Santa Clara, CA, USA). Samples with RNA Integrity Numbers (RINs) below 8 were excluded from this study. RNA purity was assessed by measuring absorbance at 230 nm, 260 nm, and 280 nm with a Nanodrop ND1000 spectrophotometer (Thermo Scientific, Villebon-sur-Yvette, France). Subsequently, mRNA was purified using the Dynabeads™ mRNA Purification Kit (Thermo Fisher Scientific, France) and quantified with a Qubit RNA assay kit (Life Technologies, Carlsbad, CA, USA).

### 3.6. Library Preparation, Sequencing, RNA-Seq Analysis, and Validation

cDNA libraries were prepared from 10 ng of mRNA using the SMARTer Stranded RNA-Seq Kit according to the manufacturer’s guidelines (Takara Bio USA, Mountain View, CA, USA). The libraries were analyzed and quantified as described in [120]. Pooled libraries were sequenced on an Illumina NextSeq500 using the Illumina Mid Output Kit V2.5 (150 cycles) to generate 75 base-pair paired-end reads. Raw sequences have been deposited in the NCBI Gene Expression Omnibus (GEO) with accession number: GSE281877 (http://www.ncbi.nlm.nih.gov/geo, accessed on 14 November 2024).

FASTQ files were imported into CLC Genomics Workbench v22, with poor-quality reads (<Q30) discarded. For each library, reads were trimmed and filtered based on the following criteria: sequence quality <0.01, up to 2 ambiguous nucleotides allowed, minimum read length >35 nucleotides, trimming against Illumina adaptor sequences, and a hard trim of 10 nucleotides at the 5′ end. The filtered reads were then mapped to the *Malus* × *domestica* GDDH13 V1.1 transcriptome [121] using stringent mapping criteria: mismatch, gap, and insertion costs set at 2, 3, and 3, respectively. Reads with more than 10 hits were removed, and only reads with 80% identity and 80% coverage to the reference transcriptome were retained. Expression values were calculated using the RPKM (Reads Per Kilobase of transcript per Million reads) method [122].

Differentially expressed genes among apple cells collected at T1, T2, T3, and T4 (n = 3) were identified using an ANOVA test [123]. A false discovery rate (FDR) corrected p-value (Benjamini–Hochberg correction) was set at 0.05, with a fold-change cut-off value of 2-fold increase or decrease in expression, and a minimum mean RPKM difference of 5 between groups. Gene ontology (GO) enrichment analysis was performed on the significantly regulated genes using Cytoscape v3.1.0 with the ClueGO v2.1.1 [124] and CluePedia v1.1.1 plugins [125], focusing on GO terms from levels 3 to 8 and a kappa score set at 0.2. Principle component analysis (PCA) and pathways analysis conducted using iDEP.96 (http://bioinformatics.sdstate.edu/idep96/, accessed on 10 March 2024) [39]. To validate the reliability of the RNA-Seq data, the expression levels of 12 randomly selected genes were measured using real-time quantitative reverse transcription PCR (qRT-PCR). Details and protocols are described in Supplementary Data S1.

### 3.7. Stastical Analysis

The statistical significance of differences in triterpene composition between fruit skins and cell cultures was evaluated using Student’s *t*-test (*p* < 0.001). For the triterpene profiles across different time points in the bioreactor experiment, a one-way ANOVA followed by Tukey’s post hoc test was conducted to identify significant differences between groups. Statistical analyses were performed using SPSS 13.0 software (SPSS Inc., Chicago, IL, USA).

## 4. Conclusions

In this study, we developed a high-yield triterpene-producing cell suspension culture, leveraging its exceptional bioactive properties. A comprehensive transcriptomic analysis of Cox cell cultures across different growth phases reveals intricate regulatory networks governing biomass production and triterpene biosynthesis. Key biological processes, including cell cycle regulation, cell wall biosynthesis, lipid metabolism, and stress responses, play crucial roles in these dynamics. These findings offer a deeper understanding of the regulatory pathways that can be used to develop targeted strategies for enhancing yield, stability, and efficiency in large-scale applications. Furthermore, this study inspires future directions that integrate bioreactor technology with metabolic engineering, paving the way for innovative industrial applications.

## Figures and Tables

**Table 1:** Pathway enrichment analysis on principle component 1 and 2 obtained from PCA using IDEP.96 [39].

Principle Component	Biological Process	e-Value
PC1	Nuclear division	4 × 10^−8^
	Organelle organization	7 × 10^−13^
	Microtubule-based process	8 × 10^−8^
	Cellular component organization	8 × 10^−8^
	Chromosome organization	4 × 10^−11^
	Cellular component organization or biogenesis	6 × 10^−11^
PC2	Translation	2 × 10^−6^
	Peptide metabolic process	4 × 10^−6^
	Peptide biosynthetic process	1 × 10^−6^
	Cellular amide metabolic process	1 × 10^−4^
	Amide biosynthetic process	5 × 10^−5^
	Organonitrogen compound biosynthetic process	2 × 10^−6^

## Data Availability

Raw transcriptomic sequences are available in the NCBI Gene Expression Omnibus (http://www.ncbi.nlm.nih.gov/geo) under accession number GSE281877.

## Supplementary Materials

The following supporting information can be downloaded at: https://www.mdpi.com/article/10.3390/ijms26073188/s1.
