# Supplementary figures and images for: Harnessing Apple Cell Suspension Cultures in Bioreactors for Triterpene Production: Transcriptomic Insights into Biomass and Triterpene Biosynthesis
Source: Int J Mol Sci. 2025 Mar 29;26(7):3188. doi: 10.3390/ijms26073188 (PMC11989043; doi:10.3390/ijms26073188)

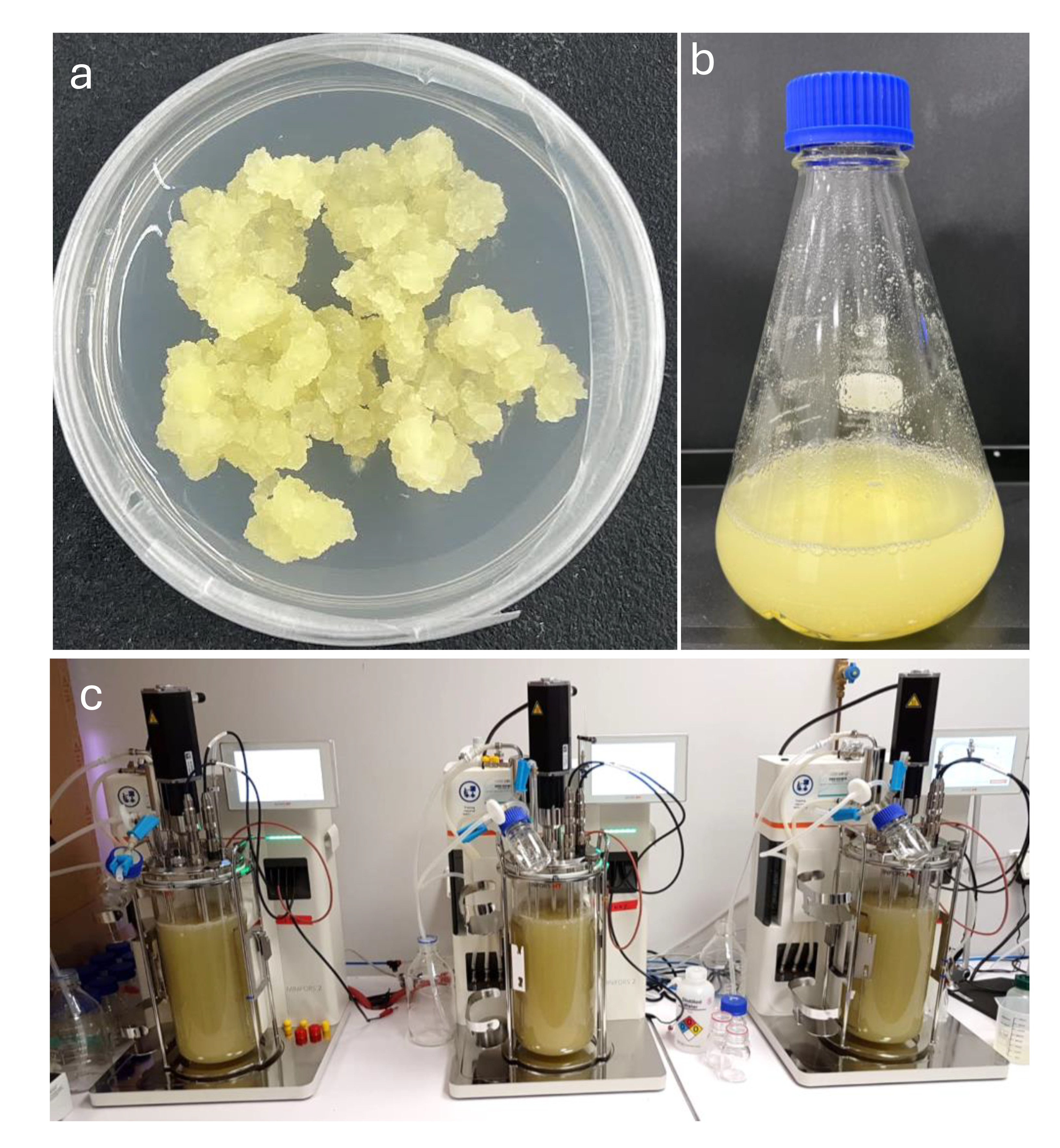

Supplement: Supplementary file 1 [file ijms-26-03188-s001.zip › FigS1.tif]
